# The Effects of Rapid Periodontal Distraction on Tooth Vitality and Bone Quality/Quantity: A Split‐Mouth Experimental Design

**DOI:** 10.1111/ocr.70143

**Published:** 2026-05-26

**Authors:** Parul Sangwan, Ying Liu, Phillip M. Campbell, Michael Ellis, Yan Jing, Peter H. Buschang

**Affiliations:** ^1^ Department of Orthodontics Texas A&M University College of Dentistry Dallas Texas USA; ^2^ Department of Biomedical Sciences Texas A&M University College of Dentistry Dallas Texas USA; ^3^ Department of Oral Maxillofacial Surgery Texas A&M University College of Dentistry Dallas Texas USA

**Keywords:** bone regeneration, orthodontic tooth movement, periodontal distraction, tooth vitality

## Abstract

**Objectives:**

Periodontal distraction (PD) osteogenesis is a minimally invasive technique capable of rapidly moving uniradicular teeth, thereby shortening orthodontic treatment time. This study aimed to determine whether: (1) PD‐induced tooth movement affects tooth vitality and (2) the regenerated bone meets restorative standards for implant placement.

**Materials and Methods:**

In six dogs, the maxillary 2nd premolars on the experimental side were extracted, the mesial inter‐radicular bone was removed to within 1 mm of the 1st premolar, and an intraoral distraction device was bonded. The contralateral side served as a negative control. After a 5–7 day latency period, the 1st premolars were distracted distally at 1 mm/day for 6 days, followed by a 3‐week consolidation period. Tooth vitality was assessed with Laser Doppler Flowmetry (LDF) and histological analysis; bone quality and quantity were evaluated by μCT.

**Results:**

Cast and intraoral measurements showed 4.7–4.8 mm of tooth movement at a rate of 0.8 mm/day. LDF recordings displayed pulsatile signals synchronized with each dog's heartbeat, indicating pulpal vitality, which was confirmed histologically by: (1) red blood cells in the pulp, (2) odontoblasts at the pulp periphery expressing Osterix, (3) rich vasculature revealed by GS‐IB4 staining, and (4) similar blood vessel density between control and experimental sides. μCT analyses revealed slightly less dense, less mature bone mesial to the 1st premolar, but with complete or near‐complete vertical and buccolingual fill. Mild root resorption was observed, with some resorptive lacunae, particularly on lingual surfaces.

**Conclusions:**

PD can be performed at rates faster than previously reported without short‐term adverse effects on tooth vitality or root integrity. Movement at 0.8 mm/day may substantially reduce treatment time in crowded, extraction cases. Additionally, PD offers a novel means to generate bone that may be suitable for implant placement in patients with congenitally missing teeth or partial edentulism.

## 1 Introduction

The orthodontist's decision to extract teeth can be a major determinant of treatment duration. On average, extraction cases take 25–35 months to finish, compared to 21–27 months for non‐extraction cases [1]. Extended treatment times lead to patient frustration and compliance burnout, as well as increased risks of developing white spot lesions, caries, gingival inflammation and root resorption [2, 3, 4]. When continuous forces are applied, teeth move at a rate of 0.8–1.2 mm/month, which is problematic when teeth have to move long distances [5, 6]. Accelerating tooth movement would allow orthodontists to treat to the same standard in less time, increase patient satisfaction and mitigate the risks involved with longer treatments.

Surgery is the most common method used to accelerate tooth movements. Surgical‐assisted orthodontics was pioneered by European clinicians who believed that cortical bone had to be reduced to move teeth rapidly. This led to the development of less invasive corticotomies [7], which produce faster tooth movements through a biological healing process called the Regional Acceleratory Phenomenon (RAP) [8]. Experimental studies have verified that corticotomies significantly increase tooth movement via the RAP [1]. Corticotomies can double the rate of tooth movement, but only within a narrow window of time limited by the rapidity of the healing response.

Periodontal distraction (PD) is a novel technique that promises to provide even faster rates of tooth movement than corticotomies. It is an alternative, less invasive application of distraction osteogenesis that may be more readily accepted by patients. Based on the hypothesis that the periodontal ligament behaves like a ‘suture’ between the tooth and alveolar bone, PD was introduced in 1998 as a surgical procedure to expedite canine retraction [9]. Experimentally, teeth have been distracted at rates of 0.25–1.0 mm/day and moved total distances of 0.9–5.0 mm [10, 11, 12, 13] (Table S1). Clinical studies, which evaluated the effects of PD on teeth distracted at rates ranging from 0.25–1 mm/day, have reported little or no root resorption [9, 14, 15, 16, 17, 18, 19, 20, 21] (Table S2). While the PDL appears to initially rupture during rapid PD, Sharpey's fibres remain attached to the tooth and alveolar bone [12] and the periodontium returns to their initial states 10 weeks post‐PD [11, 13].

Importantly, the orthodontic literature does not provide conclusive evidence concerning the effects of faster rates of periodontal distraction on tooth vitality. Laser Doppler Flowmeter (LDF) has shown that the effects of PD are similar to conventional orthodontics when teeth are moved for 4 days at 0.5 mm/day [13]. Histology has also shown that teeth moved at 0.5 mm/day remain vital [11]. Clinical studies using electric pulp testing (EPT) to evaluate vitality have reported mixed results post‐PD (Table S2). However, the effects of distracting at 1 mm/day on tooth vitality remain unclear. The one study that moved teeth at faster rates reported vitality loss in 1/3 of the teeth but did not distinguish between teeth moved at faster and slower rates [9]. Additionally, few studies have verified pulp vitality histologically. Using H&E staining, Lv et al. [11] reported hyperaemia in the pulp of dogs' teeth that had undergone PD, but they did not assess the activity of odontoblasts and blood supply.

The purpose of the present study was to evaluate the pulpal health of teeth distracted at a rate of 1 mm/day with LDF and histological assessments and to determine whether the quality and quantity of the regenerate bone are sufficient for implant placement.

## 2 Materials and Methods

The present study included six skeletally mature and healthy female hounds, approximately 15–26 months of age and weighing 68–86 pounds. Dogs are a well‐suited model for studying bone remodelling and tooth movement because their dentition and periodontium are comparable to humans [11, 22]. Dogs in this age range had attained their adult body size, making it possible to use more easily, place, and maintain traditional orthodontic appliances. In addition, adult dogs minimized the potentially growth effects that could have biased the results. One side of the maxillary 1st premolar was distracted distally, while the other side was used as the negative control with no tooth movement.

### 2.1 Pre‐Surgical Preparation

After a 10‐day quarantine, the dogs were sedated, dental prophylaxis was performed and maxillary alginate impressions were made. Occlusal and periapical radiographs were taken of each quadrant. The quadrant with the less distally angulated 1st premolar root was chosen as the experimental side. At the next appointment, the dogs were sedated and burs were used to lengthen the crown by removing the gingiva and bone around the 1st premolar, which increased the surface area for bonding the distraction device and aided in the retention of the distraction device (Figure S1a). Periapical radiographs were taken to verify adequate bone removal and alginate impressions were taken to fabricate the appliances.

### 2.2 Appliance Design

The maxillary impressions were poured in die stone and orthodontic band material was adapted and welded to fit the maxillary canine, 1st premolar and 3rd premolar. A Herbst tube 0.072″ in diameter was soldered to the 3rd premolar band. The arm of a 12 mm mini rapid palatal expander was soldered to the canine band and inserted through the Herbst tube on the 3rd premolar band. 0.045″ orthodontic wire was soldered to the 1st premolar and RPE arm (Figure S1b).

### 2.3 Surgery

To prevent surgical root damage, a periapical radiograph was taken to determine the length of the 1st premolar root and to visualize the apex (Figure S2a). The maxillary 2nd premolar was then hemisected, elevated and delivered via forceps (Figure S2b). Bilateral baseline tooth vitality readings of the maxillary 1st premolars were recorded with Laser Doppler Flowmetry (LDF) for 1 min. The LDF probe was isolated from the surrounding periodontium with nitrile material to avoid contaminated signals, stabilized and oriented down along the tooth's long axis.

The PD osteotomies outlined by Liou et al. [9] were performed through the extraction site under a constant spray of sterile saline solution. Interseptal bone distal to the 1st premolar was reduced to 1 mm with a 44C surgical bur. The inter‐radicular bone of the 2nd premolar was removed to allow for 6 mm of distraction. The depth of the osteotomy was extended 1–2 mm apical to the root apex to prevent osseous interferences. The buccal and lingual walls of the cortex were left intact, but the lingual cortex was thinned. A surgical guide was moved back and forth within the osteotomy site to identify bony interferences that needed to be removed (Figure S2c).

To weaken the interseptal bone distobuccally and distolingually, undermining grooves were made to the depth of the osteotomies (Figure S2d). These oblique osteotomies connected with the vertical osteotomies and extended approximately a quarter of the way around the 1st premolar. After periapical radiographs confirmed adequate bone removal, the surgical site was irrigated with a NaCl solution and closed with 3.0 Vicryl sutures.

### 2.4 Appliance Delivery, Latency, Appliance Activation and Euthanasia

The appliance bands were perforated with a small round bur to aid in retention. After pumicing and etching with 37% phosphoric acid for 15 s, the distraction device was cemented in place with 3M Glass Ionomer cement (Figure S1c,d). The distractor was turned 3X to verify tooth movement and then turned back. Small notches were drilled into the canine and 1st premolar for daily intraoral calliper measurements. Antibiotics and analgesics were given to the dogs post‐surgery, and there were chlorhexidine gluconate rinses daily for 7 days.

After a 5–7 day latency period, the 1st premolar was distracted distally at a rate of 1 mm/day for 6 days. A 3‐week consolidation period followed to allow for bone remodelling, after which the bands were sectioned and peeled off from the teeth. This protocol has been previously shown to be sufficient for angiogenesis and new bone formation in experimentally created wound compartments [23] and extractions sites [24]. Final records consisted of periapical radiographs, intraoral photographs, alginate impressions and LDF recordings. After surgical plane anaesthesia was confirmed, the carotid arteries were exposed and cannulated, and the external jugular veins were severed. 2 cc of Beuthanasia‐D was injected intracardially and cessation of heart function was confirmed with a stethoscope. The specimens were harvested and fixed with 4% paraformaldehyde (pH 7.4).

### 2.5 Measurements of Tooth Movement and Vitality

All measurements were made bilaterally. Three intraoral calliper measurements of the distance between the canine and 1st premolar were made daily and averaged. In addition, the distance between the mesial of the canine to the middle of 1st premolar was measured on the pre‐PD and post‐PD dental casts. 1st premolar tipping was measured on the pre‐PD and post‐PD radiographs using Viewbox 4.0 software (Figure S3).

The pre‐PD (after anaesthesia but prior to surgery) and post‐consolidation LDF recordings were analysed using Fast Fourier Transform (FFT). Analyses were performed on two different 10‐s intervals. A tooth was considered vital if a peak in the signal coincided with the dog's heartbeat.

### 2.6 Radiological Assessment of Bone and Root Resorption

The samples were placed in 30 mm wide μCT tubes filled with 0.5% paraformaldehyde, sealed with parafilm and scanned with a Scanco μCT 35 scanner at 30 μm resolution, using 55 kVp, 145 μA settings and an 800 ms integration time. Analyze V12.0 software was used to visualize root resorption, the size and quality of the regenerate bone mesial to the 1st premolar. Three‐dimensional reconstructions were used to measure bone width and height. Width was measured at the widest portion of the bony ridge in the middle portion between canine and 1st premolar. Height was measured as the distance from the surface at the lowest point of the regenerate bone to the line of the alveolar crest between canine and 1st premolar. The volumes of interest (VOI) included the lamina dura (a cylinder with 1 mm diameter adjacent to the root surface) and the trabecular bone (a cylinder with 1 mm diameter in the centre of the regenerate area), both of which were measured along the middle 80% of the root. Bone density, bone volume/total volume, as well as the trabecular number, thickness and separation, were evaluated. Side differences in width and height were classified as minimal (0–2 mm), moderate (2–4 mm) or severe (> 4 mm).

μCT images were also used to qualitatively evaluate the root surface for resorptive lacunae. The lengths of the 1st and 3rd premolar roots were measured on the initial and final periapical radiographs relative to the horizontal reference line defined by the alveolar crest. Changes in the lengths of the 3rd premolar line were used as correction factors for changes of the 1st premolar line.

### 2.7 Histologic Analysis

Following μCT analysis, the samples were decalcified with EDTA, dehydrated in graded alcohol, cleared with xylene, infiltrated and embedded in paraffin. They were sectioned along the sagittal plane to include the 1st premolar root, bone 3 mm apical to the root apex and the regenerate bone mesial to the 1st premolar. To illustrate pulp vitality at the cellular level, Haematoxylin and Eosin (H&E), GS‐IB4 staining (Invitrogen I21411, 1:100) [22], Osterix (Abcam ab22552, 1:100) [25] and Periostin (R&D AF2955, 1:100) IHC were performed. A bright light microscope was used for H&E and IHC, and fluorescent cell images for GS‐IB4 were captured with the SP8 Leica confocal microscope with wavelengths from 488 (green) μm [26] and used for vessel density quantitation [27]. Each pulp was first divided into coronal, middle and apical third, and four 20× views were randomly selected in each portion. The thickness of acellular (in the top portion of the root) and cellular cementum (in the middle portion of the root) was measured on the mesial and distal sides, respectively, using H&E‐stained sections. For each cementum type, 10 measurement points were randomly selected, and the mean of these measurements was used as the representative value.

### 2.8 Statistical Analysis

The statistical analyses for this study were conducted using IBM SPSS version 23 (IBM Corp., Armonk, NY, USA). All the data except the number of blood vessels were normally distributed, which allowed paired *t*‐tests to be used to assess changes over time and between‐side differences. Wilcoxon signed ranks tests were used to evaluate side differences in the number of blood vessels. One‐way ANOVA was used to analyse the thickness of acellular and cellular cementum.

## 3 Results

Following the PD surgeries, the dogs healed normally with no signs of infection or swelling.

### 3.1 Tooth Movements and Tipping

Based on the calliper and cast measurements, the experimental teeth were distracted 4.7–4.8 mm, at a rate of 0.8 mm/day (Table S3), with statistically significant between‐side differences. Daily tooth movements were similar throughout the 6‐day distraction period (Figure 1). The 1st premolars tipped 12.8°, which was significantly more than the control premolars (Table S3).

**FIGURE 1:**
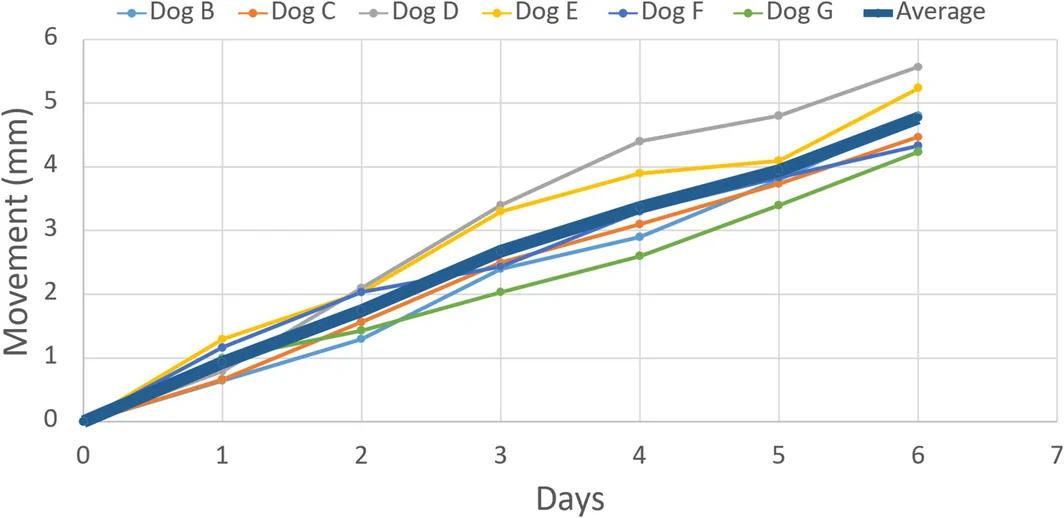
Daily tooth movements were measured intraorally over the 6‐day distraction period.

### 3.2 LDF Power Frequency Signals

The peaks of the pulsatile signals from the pulpal tissue were consistent with the dogs' heartbeats. The power of the pre‐distraction FFT signals was 0.057 and 0.058 units on the control and experimental sides, respectively, with no statistically significant between‐side difference. At the end of the consolidation period, the FFT signals were 0.033 PUs and 0.042 PUs, respectively, again showing no statistically significant between‐side difference (Figure 2).

**FIGURE 2:**
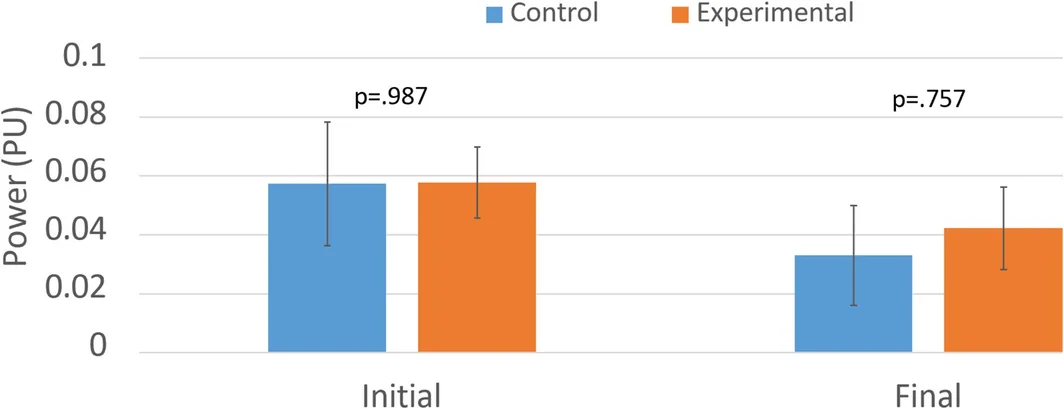
Initial (Day 0) and final (post‐consolidation) power of the peak frequency signals were assessed with LDF on the control and experimental premolars.

### 3.3 Histology and IHC

H&E staining showed that loose connective tissue was evenly distributed in the pulp cavity, with no sign of necrosis on the experimental side (Figure 3, left panel). There were many vessels with red blood cells in the pulp (Figure 3, middle panel). GS‐IB4 staining also showed well‐distributed vasculature on both the central part of the pulp and the odontoblast layer of the experimental teeth, similar to the control side (Figure 4a). The quantitative analysis showed no statistically significant between‐side differences in the number of blood vessels in the coronal, middle or apical portion (Figure 4b). The polarized odontoblasts were well arranged at the outermost layer of the pulp (Figure 3, right panel) and actively expressing Osterix, a transcriptional factor for odontoblast differentiation and dentine formation (Figure S4). These data showed that, on the cellular level, the pulp of all the experimental teeth was vital.

**FIGURE 3:**
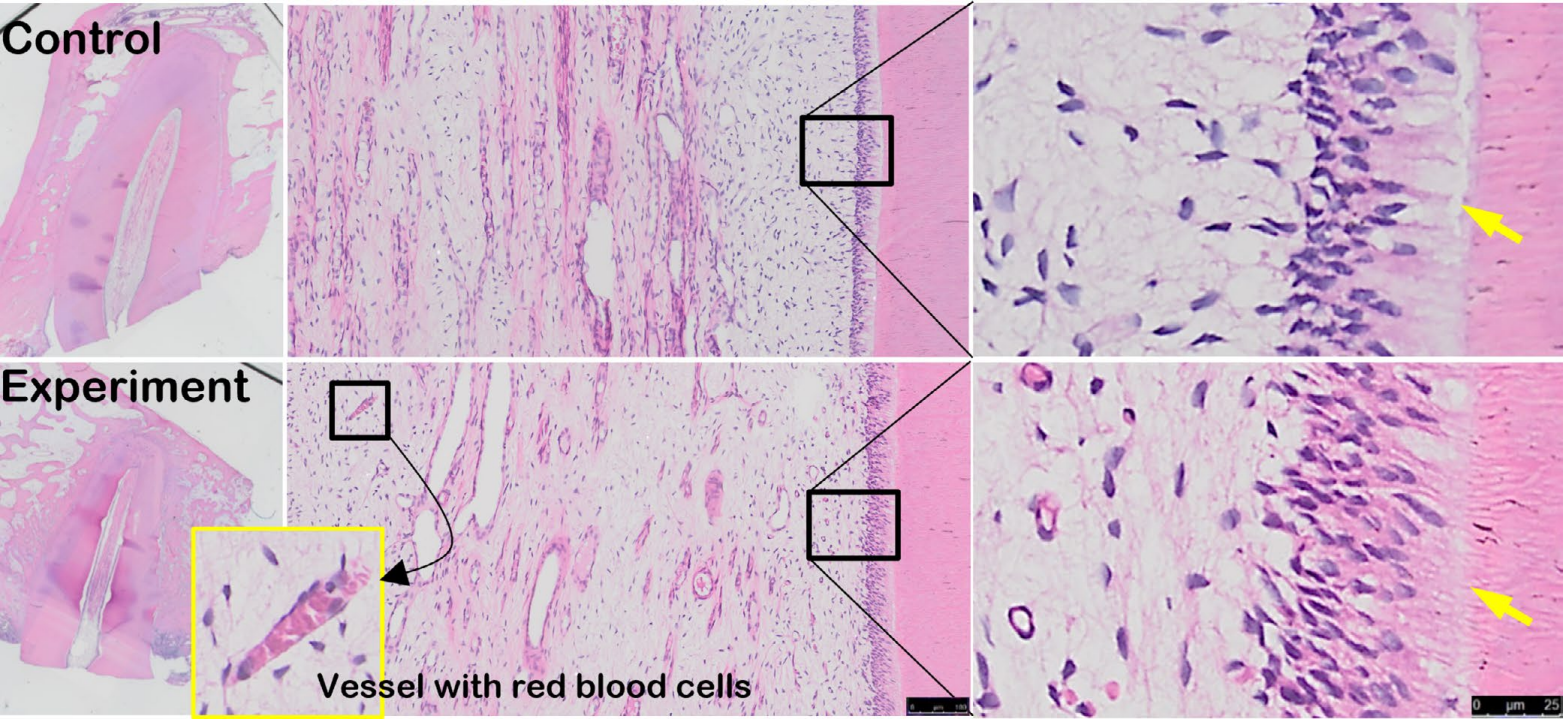
H&E staining showed numerous blood vessels (middle panel) with well‐organized odontoblast (right panel, arrows) in both experimental and control pulp.

**FIGURE 4:**
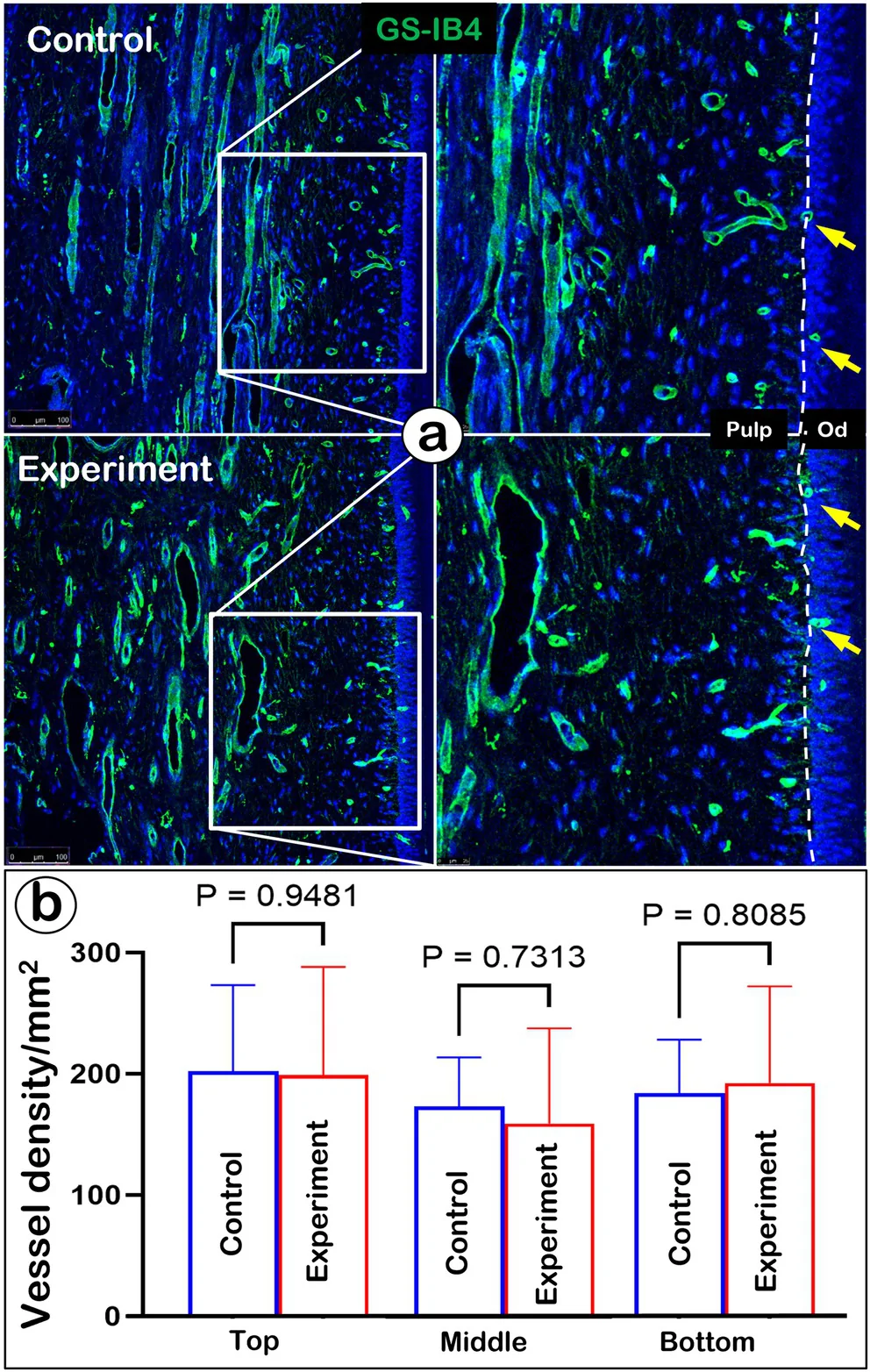
(a) GS‐IB4 staining in green showed well‐distributed vasculature in the central pulp and odontoblast layers (arrows) of both experimental and control teeth. (b) The quantitative analysis revealed no statistically significant between‐side differences in vessel density in the coronal, middle or apical portions. Od, odontoblast.

Compared to the PDL on the compression and tension side from the control groups (Figure 5a1,b1), the PDL in the experimental teeth was overall wider, with higher cell density, abundant collagen fibres and evenly distributed extracellular matrix on both sides (Figure 5c1,d1). In addition, Periostin, a matricellular protein which is highly expressed in PDL, was widely expressed in both the compression and tension sides of the experimental teeth (Figure S5).

**FIGURE 5:**
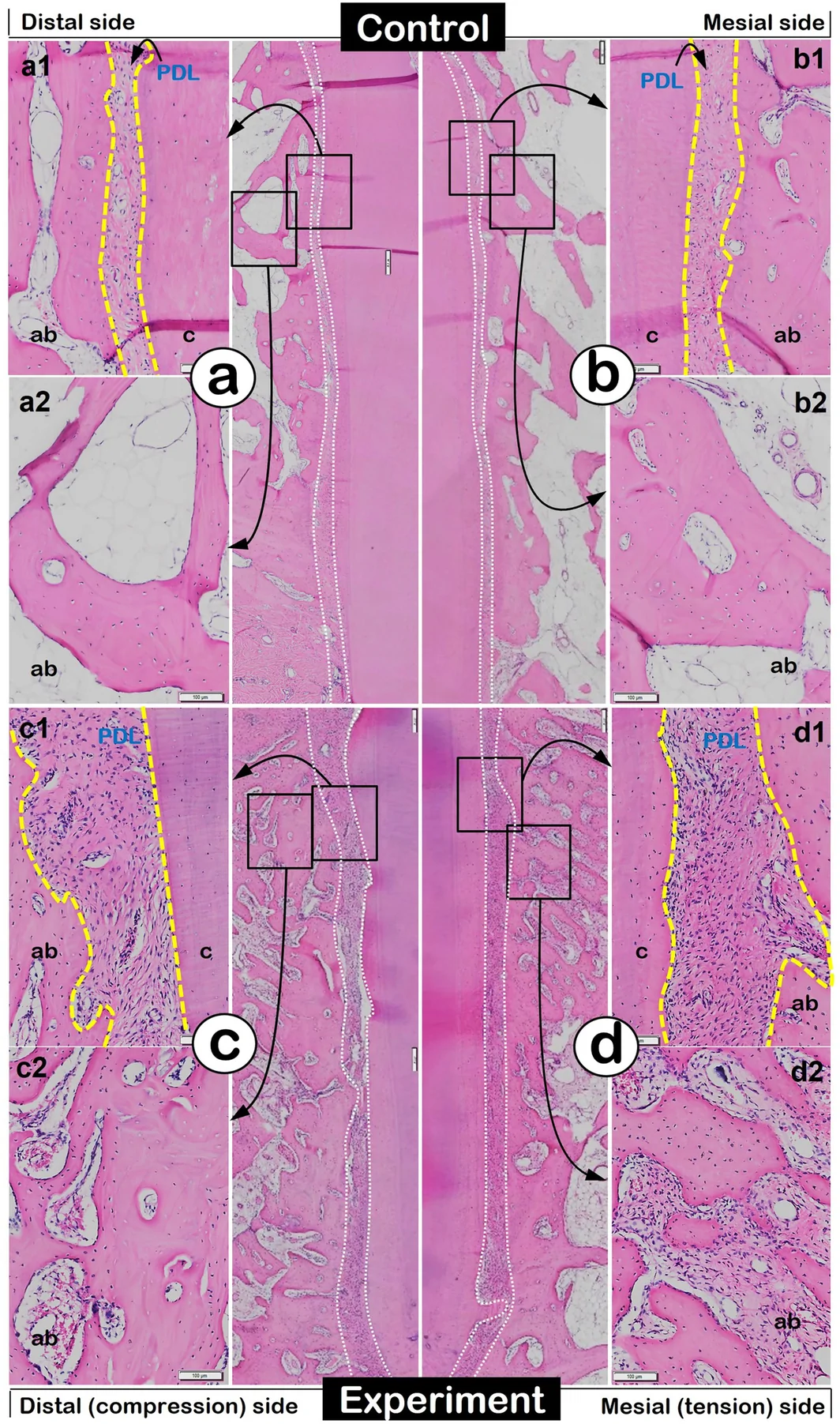
(a, b) H&E staining of control teeth showed moderate fibroblast density in the PDL on both the compression (a1) and tension (b1) sides. Several trabeculae were observed in the alveolar bone adjacent to the PDL, with a thin osteoblast layer on the surface and some embedded osteocytes on both the compression (a2) and tension (b2) sides. (c, d) In experimental teeth, the PDL was overall wider with denser fibroblasts on both the compression (c1) and tension (d1) sides. Active bone formation was evident adjacent to the PDL on both sides, with numerous osteoblasts and osteocytes within trabeculae, surrounded by active bone marrow tissue on both the compression (c2) and tension (d2) sides. ab, alveolar bone; c, cementum.

### 3.4 Root Resorption

Qualitative μCT assessments showed that every tooth had resorptive lacunae (Table S4). Root resorption was evident on the lingual surfaces of all experimental teeth and almost all of the buccal surfaces (Figure 6a). The mean difference in 1st premolar root length between the initial and final periapical radiographs was 0.2 ± 0.5 mm, which was not statistically significant (Figure 6b). Quantitative analysis revealed no significant differences in acellular cementum thickness between mesial and distal sides within each group, nor between corresponding sides across groups (Figure 6c, upper). A similar pattern was observed for cellular cementum thickness, although the mesial side of the experimental group exhibited a significantly greater thickness compared to its distal side (Figure 6c, lower).

**FIGURE 6:**
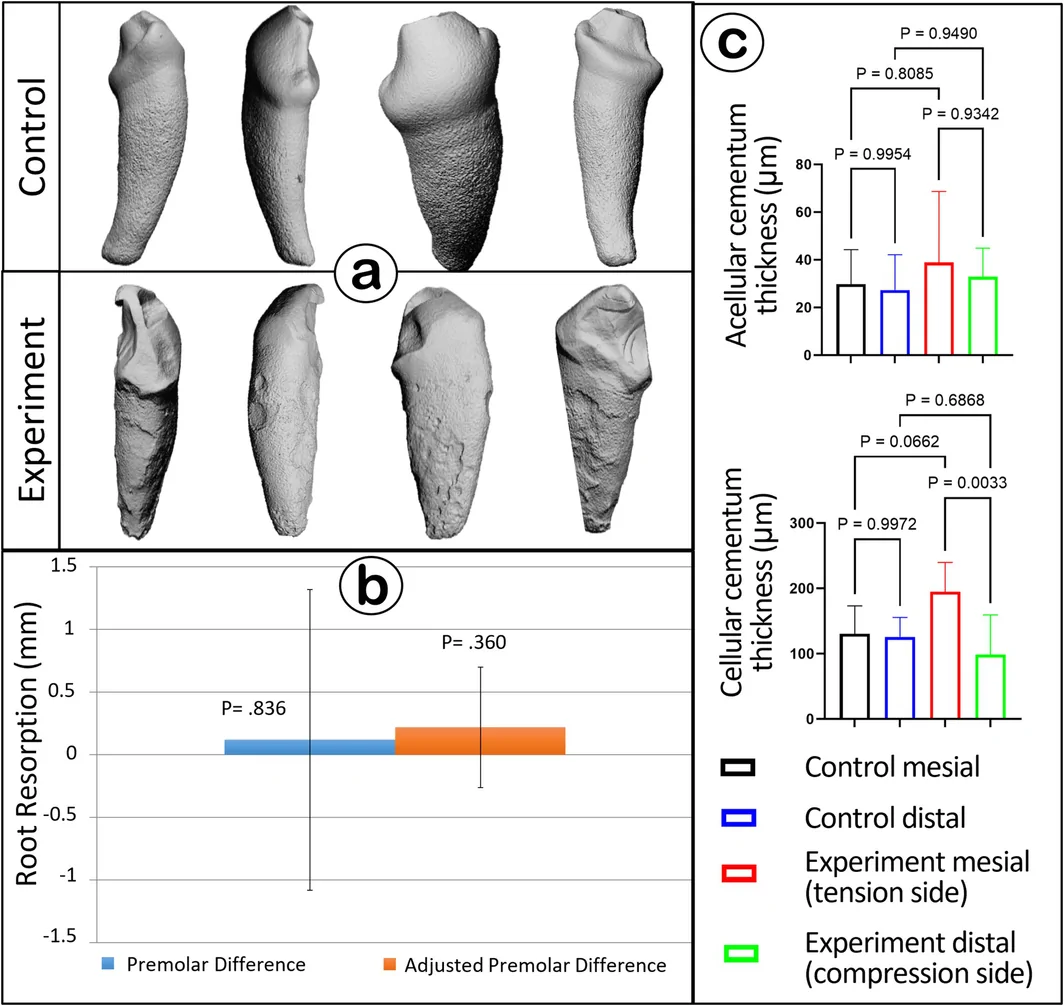
(a) μCT images showed smooth root surfaces on control root surfaces whereas resorptive lacunae were evident on the experimental teeth. (b) Apical root resorption was reflected by the actual differences in root length measurements over time (left) and the differences adjusted based on differences in 3rd premolar length over time (right). (c) Quantitative analysis of acellular (upper panel) and cellular (lower panel) cementum thickness on the mesial and distal sides of both control and experimental specimens revealed similar trends.

### 3.5 Bone Regenerate

The bone volume fraction, apparent density and material density of the lamina dura were all less on the experimental than control sides, but only material density and trabecular thickness differed significantly (Table S5). Comparisons of trabecular bone adjacent to the lamina dura demonstrated only material density had a statistically significant between‐side difference (Table S6). μCT imaging demonstrated that the regenerated bone exhibited comparable height and width to the contralateral control side (Figure S6a). Quantitative analyses confirmed that there were no statistically significant differences in bone heights or widths between sides (Figure S6b). All experimental dogs showed increased width with no more than 0.4 mm decreased height.

On histological level, the trabeculae of the alveolar bone adjacent to PDL in the control group were thick with moderate osteoblasts lining on the bone surface (Figure 5a2,b2). In the experimental group, both sides exhibited more numbers of alveolar trabeculae than the control (Figure 5c,d). The enlarged images further showed higher level of marrow activity with more osteoblasts lining on the bone surface and osteocyte in bone matrix, indicating active bone regeneration on both sides (Figure 5c2,d2). The trabeculae were lined parallel on the tension side, indicating new bone formed along with the direction of distraction force.

## 4 Discussion

Rapid tooth movement with periodontal distraction at a rate of 1 mm/day does not cause loss of tooth vitality. Vitality in the present study was confirmed histologically based on the presence of red blood cells, similar vessel density in pulp, active odontoblasts, as well as evidence of dentine formation. Moreover, the FFT analyses of LDF recordings showed pulsatile signals in the pulpal tissues consistent with the heart rate of each dog, and there were no between‐side differences in the power of the signals. In addition, pulpal blushing was seen in one dog after consolidation, demonstrating blood flow (Figure S7).

EPT performed 12 months post‐distraction has shown vitality of teeth 0.5 mm/day [16]. Studies that distracted teeth at rates similar to those in the present study reported EPT vitality loss for 35% [9] and 31% [21] of teeth immediately or 1 month after distraction, respectively. Importantly, EPT is limited in its ability to assess tooth vitality during treatment because it tests nerve function (from peripheral Aδ fibres) rather than blood flow to the pulp. Aδ fibres are particularly susceptible to oxygen deprivation and can lose function during traumatic tooth movement, even though the pulp still has blood flow and is vital, which is why EPT cannot distinguish between reversible and irreversible pulpitis [28]. Even orthodontic forces elevate the threshold response of EPT, with effects lasting up to 9 months [29].

Pulpal blood flow assessed with LDF decreases to 70% of baseline readings after 4 days of distraction at 0.5 mm/day, compared to 80% with conventional orthodontic mechanics [10]. The same study showed no significant differences in odontoblastic apoptosis and differentiation between PD and conventional orthodontics.

Few PD studies have verified the pulp vitality on a histological level. Lv et al. reported hyperaemia in the PD teeth, which was not found in the present study [11]. The present study used multiple assays, including H&E, GS‐IB4 staining, the Osterix IHC and vessel density in pulp to show that pulp vitality was largely unaffected by PD. Both the experimental and control teeth had well‐organized connective tissue in the pulp, with polarized odontoblasts and rich vasculature. In summary, the present study indicates that short‐term PD at a rate of up to 1 mm/day is compatible with maintaining pulpal health.

In addition, PD has minor effects on the health of PDL. The PDL of experimental teeth on both compression and tension sides had well‐organized PDL cells and fibres with no sign of avulsion. The strong and even expression of Periostin confirmed the integrity and function of PDL in the experimental group. These data provide promising biological evidence for the future clinical applications of PD. The widened PDL observed in the experimental teeth is likely a secondary effect of the Regional Acceleratory Phenomenon (RAP), in which increased bone turnover and transient osteopenia alter the mechanical properties of the alveolar bone [8, 30]. This, combined with ongoing periodontal ligament remodelling which can last for weeks after orthodontic treatment [31, 32], may result in a temporarily expanded PDL space during the post‐movement consolidation phase.

In the present study, PD distracted teeth at a rate of 0.8 mm/day, which represents 80% of distractor activation. At a rate of 0.8 mm/day, PD can close 7 mm extraction sites in 9 days rather than 6–7 months. The PD literature shows that the total amount of distractor activation is more than the amount of tooth movement that occurs (Tables S1 and S2). In addition, 1 mm/day represents the upper limit of periodontal distraction rates reported in the literature, with limited evidence regarding the safety of higher rates.

Tipping should be expected when rapidly moving teeth with PD. In the present study, the distracted teeth tipped 12.8° distally. Tipping does not appear to be related to the rate of distraction. Studies moving teeth 0.75 mm/day or more [12, 14, 21] reported 7.2°–21.9° of tipping, while those that distracted at rates of 0.25–0.5 mm/day reported 5.3°–16.5° of tipping [12, 13]. Tipping occurs because the distractor applies force occlusal to the tooth's centre of resistance. Tipping could be advantageous when moving teeth with heavy forces because it results in less apical displacement and, consequently, less trauma to the pulpal blood supply [9].

After 3 weeks of consolidation, PD at a rate of 1 mm/day produced regenerate bone that was slightly less dense and less mature than control bone. In the present study, there was a significant difference in material density, indicating that the regenerate bone was less mineralized and that woven bone may still be present. A mixture of woven and lamellar bone has been reported in the alveolus after 6 weeks of healing [23]. The between‐side trabecular bone differences also indicated less mature bone on the distracted side. Mature alveolar bone should have fewer, thicker and sparser trabeculae [33], which was confirmed by H&E images (Figure 5). Full alveolar bone maturation can take up to 8–12 weeks [33].

The present study is the first to use μCT to evaluate the quality and quantity of the regenerate bone formed from PD. Previous μCT analyses of bone allowed to consolidate for 6 weeks after dentoalveolar distraction also showed less dense and less mature regenerate than control bone [34, 35]. The reparative and remodelling phases of bone healing, from callus formation to remineralization, require 1–4 months [36]. After distraction, the vertical and buccolingual dimensions of regenerate bone in the present study were comparable to the untreated bone. After experimental dentoalveolar distraction, complete or almost complete bone regeneration in the vertical and buccolingual dimensions has been reported [34, 35]. Based on 2D and 3D analysis, the bone fill in the present study provides sufficient dimension (Figure S6) and structural quality (Tables S5 and S6) that meet the restorative guidelines for implant placement [37, 38], suggesting PD as a method for retracting single‐rooted teeth for restorative prosthetics.

One limitation of this study is the absence of control groups representing conventional orthodontic tooth movement and osteotomies without tooth movement. Future studies incorporating these controls are needed to further characterize bone regeneration during PD at the histological level and to systematically compare bone remodelling dynamics by analysing osteogenesis and osteoclastogenesis among different orthodontic tooth movement strategies.

PD in the present study produced a number of resorptive lacunae, but no appreciable apical root resorption. Quantitative analysis further demonstrated that the thickness of both acellular and cellular cementum was comparable between mesial and distal sides within or between groups, except that the cellular cementum on the mesial side of the experimental group was significantly thicker than its distal side (Figure 6). Collectively, these findings indicate that PD did not induce severe root resorption. Several clinical studies reported little or no root resorption and no ankylosis after PD [9, 10, 16, 39]. This could be due to the short treatment duration; longer durations have been linked to the increased incidence of root resorption among orthodontic patients [40, 41]. The surgical procedure also undermines interseptal bone and removes osseous interferences that could have produced pressure‐induced root resorption. μCT evaluations of the present study showed resorptive lacunae on all lingual root surfaces and almost all buccal root surfaces. Resorptive lacunae have previously been reported 2 weeks after dog teeth had been distracted 0.5 mm/day for 2 weeks, with partial repair after 8 weeks [11]. Other studies suggest that lacunae repair starts while forces are being applied [42, 43]. The lacunae can be explained by the anatomy of dog jaws and the rigidity of the appliance. The alveolar ridge of dogs is slightly curved (Figure S2) and the appliance distracts linearly, making it likely that the teeth still experienced pressure‐induced root resorption, especially on the lingual surface.

In summary, this study demonstrates that PD, as a minimally invasive surgical procedure that can easily be completed at the time of extractions to move teeth rapidly, could reduce the need for skeletal anchorage and mitigate the iatrogenic side effects associated with longer treatment durations. PD could also be used to distract single‐rooted teeth and create sufficient bone for implant placement in edentulous patients, as well as patients with congenitally missing teeth.

## 5 Conclusions

1. Periodontal distraction at a rate of 1 mm/day causes single‐rooted teeth to move at a rate of 0.8 mm/day.
2. Periodontal distraction at a rate of 1 mm/day does not cause short‐term loss of tooth vitality.
3. After 3 weeks of consolidation, the regenerate bone produced with periodontal distraction was slightly less mature and less dense than control bone; however, within the limitations of this study, it demonstrated sufficient dimensional characteristics and favourable structural properties to support periodontal health and may be suitable for implant placement.
4. Periodontal distraction at a rate of 1 mm/day causes mild root resorption.

## Author Contributions

**Parul Sangwan:** methodology, data collection, formal analysis, original draft. **Ying Liu:** methodology, data collection, formal analysis. **Phillip M. Campbell:** conceptualization, project administration, validation, supervision. **Michael Ellis:** conceptualization, methodology, data collection, project administration, validation, supervision. **Yan Jing:** project administration, supervision, validation, methodology, data collection, formal analysis, review and editing. **Peter H. Buschang:** conceptualization, formal analysis, funding acquisition, methodology, project administration, supervision, validation, original draft, review and editing.

## Funding

Research was partially funded by the Robert E. Gaylord Endowed Chair in the Department of Orthodontics, Texas A&M College of Dentistry.

## Ethics Statement

The project was approved by the Texas A&M University College of Dentistry Institutional Animal Care and Use Committee (IACUC 2018‐0257‐CD0).

## Consent

The authors have nothing to report.

## Conflicts of Interest

The authors declare no conflicts of interest.

## Supporting information


**Figure S1:** (a) Intraoral images showed before (left) and after (right) crown lengthening for the maxillary 1st premolar (arrow). (b, c) Distraction device was fabricated on a stone cast (b) and cemented intraorally (c). (d) X‐ray was taken to confirm that the distraction device was cemented in place.
**Figure S2:** (a) Root length measurement was used to determine the site of surgical cut on the buccal surface needed to visualize the root apex. (b) The maxillary 2nd premolar was extracted. (c) Surgical guide was used to ensure sufficient vertical and transverse space for tooth distraction. (d) Oblique undermining grooves were made to weaken the interseptal bone distobuccally and distolingually (yellow arrows).
**Figure S3:** A horizontal reference line (yellow) was drawn from the alveolar crest distal of the canine to the alveolar crest mesial to the 3rd premolar; vertical reference lines (red) were drawn through the long axes of the 1st and 3rd premolars. Tipping of the 1st premolar was calculated based on angular changes (red arrow) over time. Changes in 3rd premolar angulation (yellow arrow) over time were used as adjustment factors for radiographic distortion.
**Figure S4:** IHC showed a high expression of Osterix (Osx) in the odontoblasts in the teeth.
**Figure S5:** IHC images showed Periostin was evenly expressed on the compression and tension sides of control (a1, a2) and experimental teeth (b1, b2). ab, alveolar bone; c, cementum.
**Figure S6:** μCT imaging demonstrated that the regenerated bone exhibited comparable height (red arrow heads in the upper panel) and width (red double arrows in the lower panel) to the contralateral control side (a). Quantitative analyses confirmed that there were no statistically significant between‐side differences in bone width or height (b) of post‐consolidation regenerate and control bone.
**Figure S7:** Pulpal blushing (arrow) of one experimental tooth indicated tooth pulp vitality.

**Table S1:** Experimental literature evaluating periodontal distraction and pulpal vitality. Only two animal studies have tested pulpal vitality after PD.
**Table S2:** Clinical studies evaluating the effects of PD on tooth movements, root resorption and pulp vitality.
**Table S3:** Total 1st premolar movements and tipping over the 6‐day experimental period.
**Table S4:** μCT qualitative assessment of the presence (X) or absence (O) of resorptive lacunae on the mesial, distal, buccal and lingual surfaces of the 1st premolar roots.
**Table S5:** μCT analysis of lamina dura adjacent to the 1st premolar.
**Table S6:** μCT analysis of the trabecular bone adjacent to the lamina dura of the 1st premolar.

## Data Availability

The data that supports the findings of this study are available in the Supporting Information of this article.
