# Supplementary material for: The Effects of Rapid Periodontal Distraction on Tooth Vitality and Bone Quality/Quantity: A Split‐Mouth Experimental Design
Source: Orthod Craniofac Res. 2026 May 26;29(6):1006–16. doi: 10.1111/ocr.70143 (PMC13752665; doi:10.1111/ocr.70143)

Figure S1

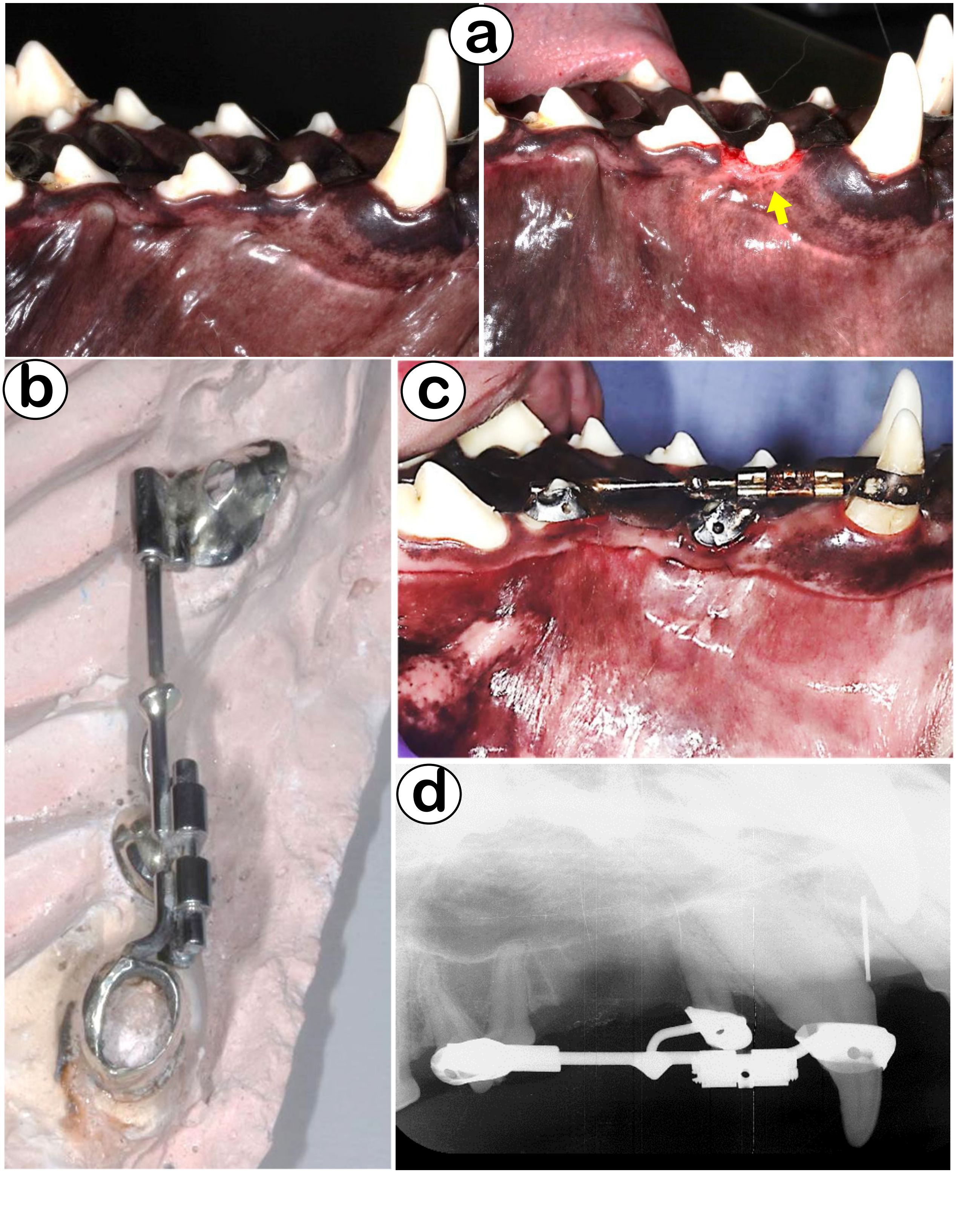

Figure S2

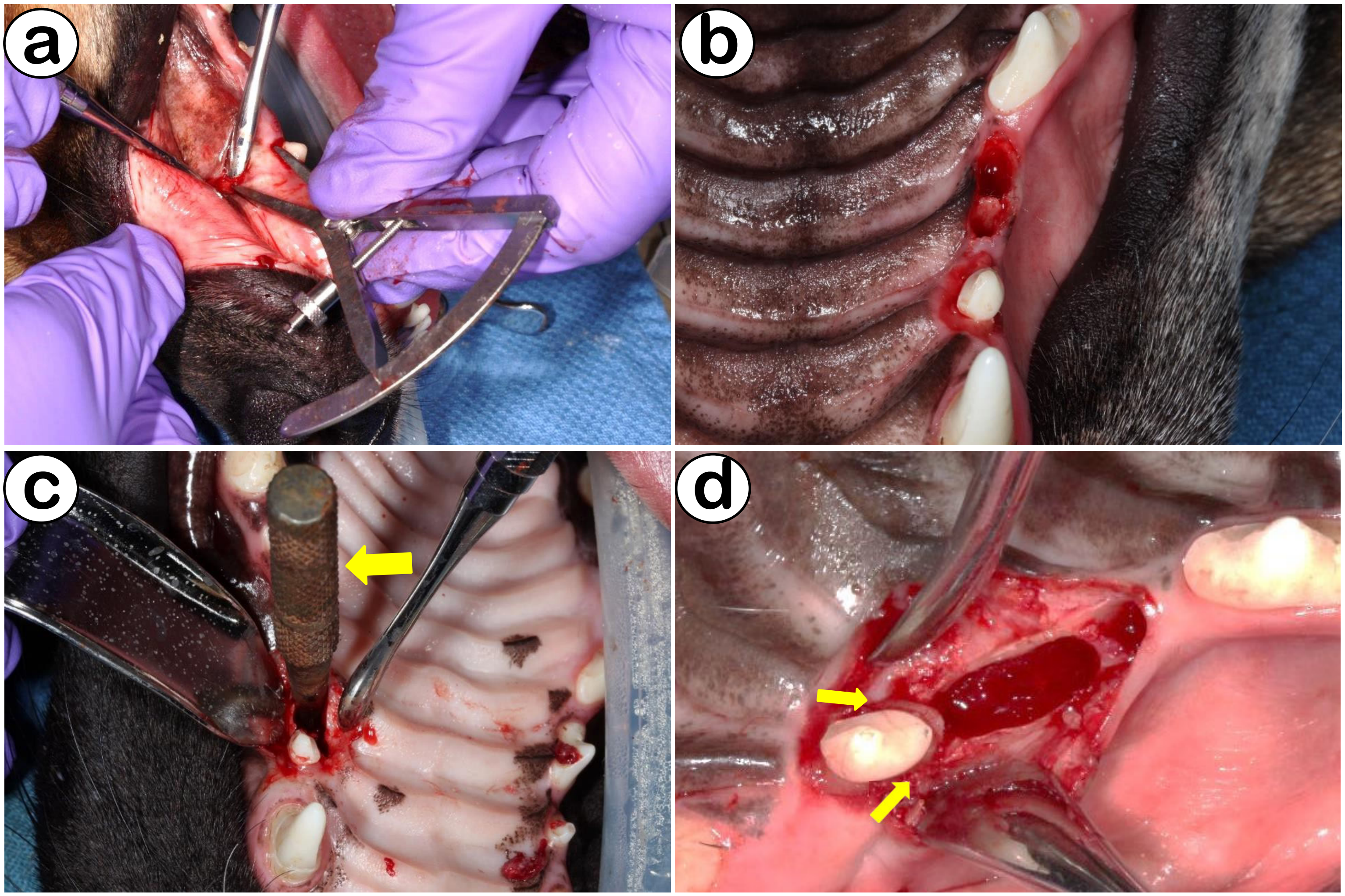

Figure S3

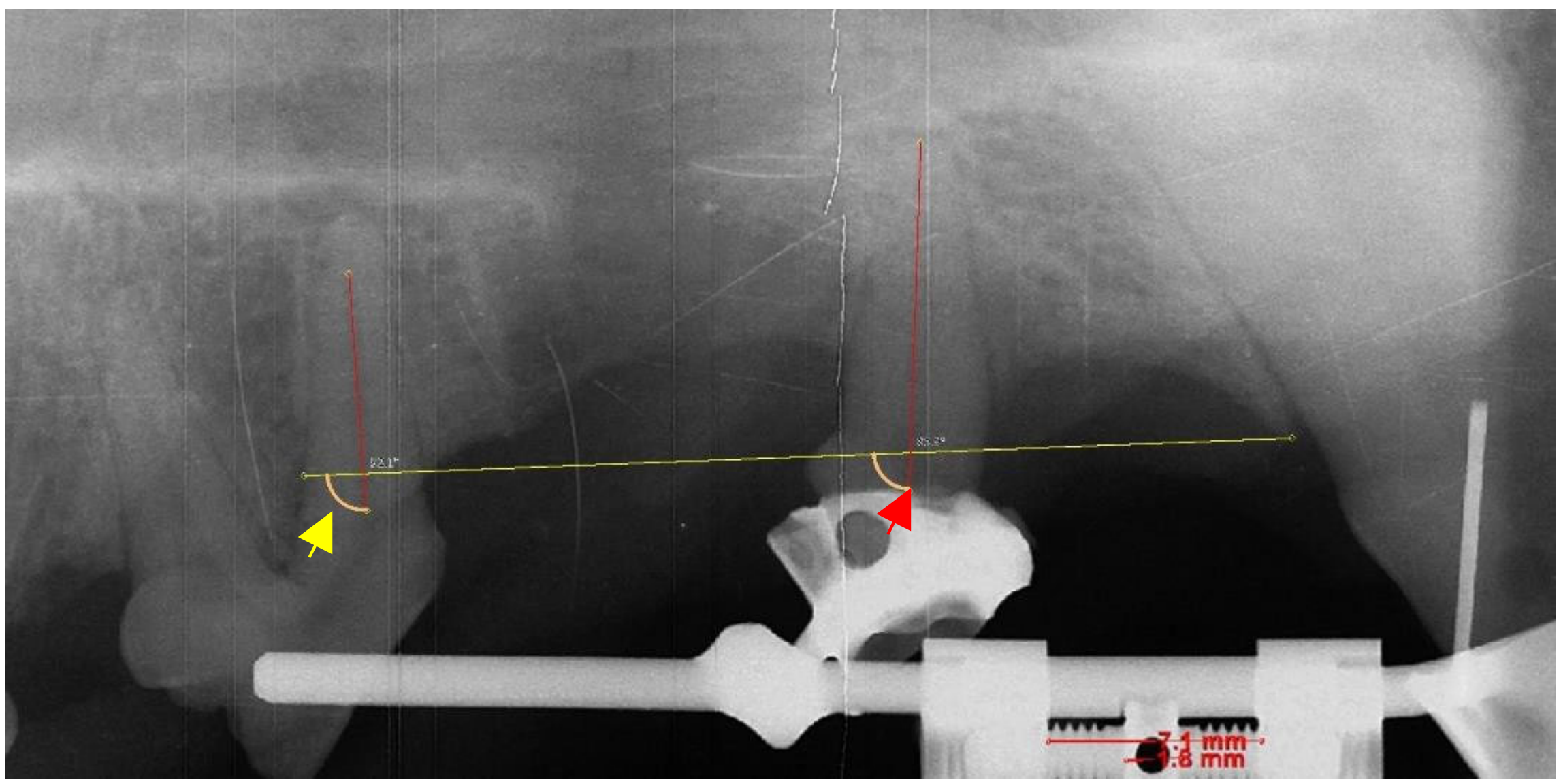

Figure S4

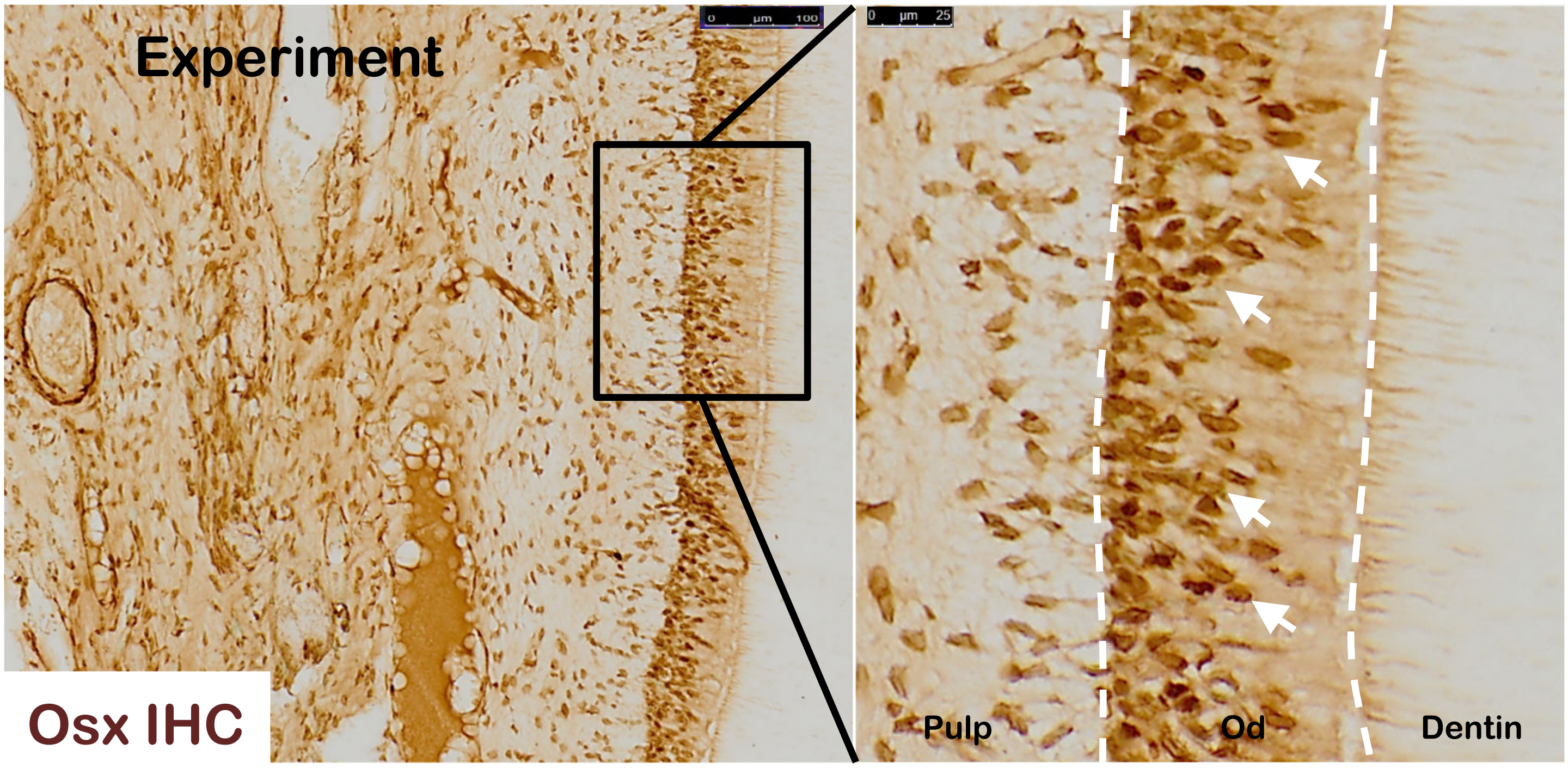

Figure S5

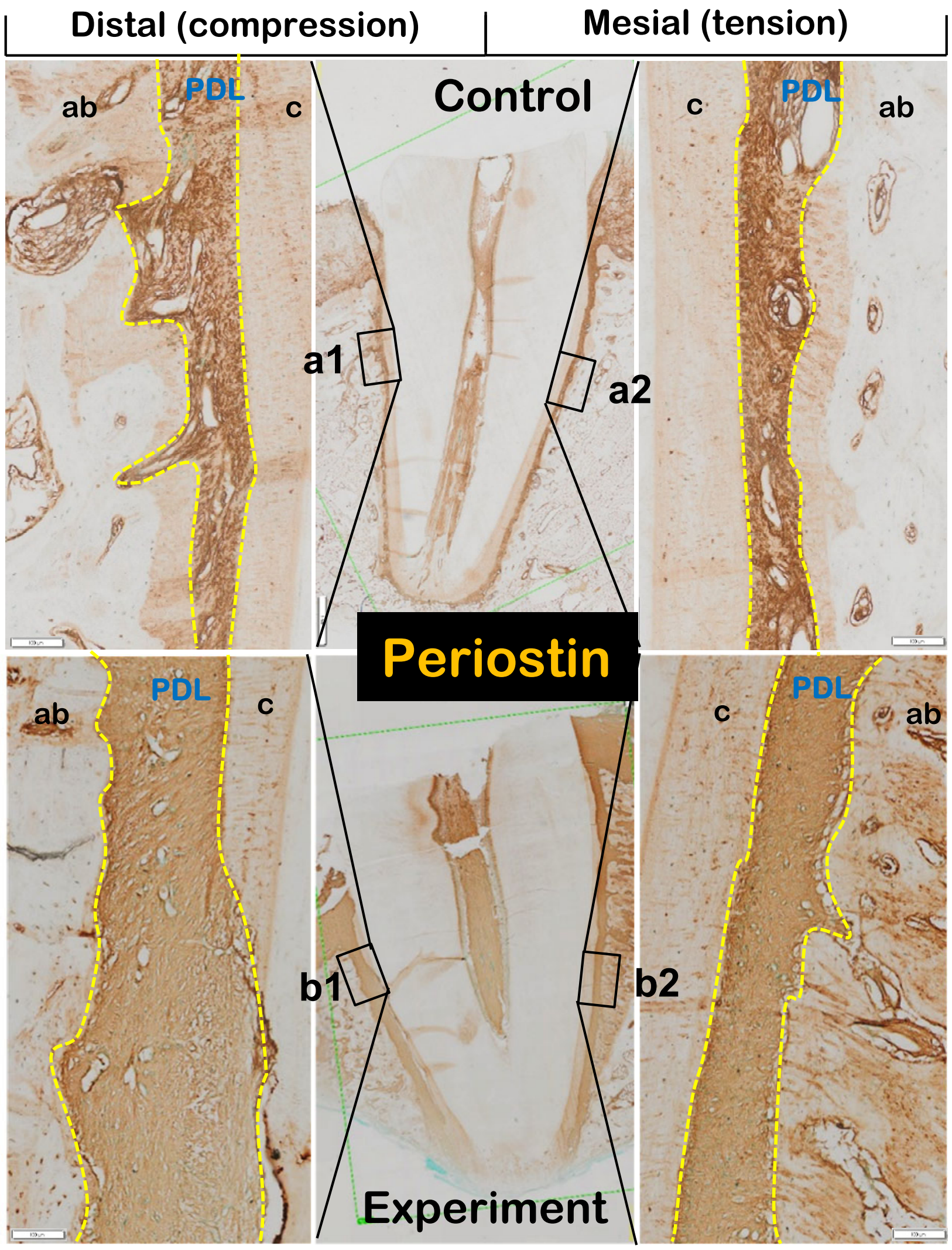
Distal (compression)
Mesial (tension)
ab
PDL
c
Control
c
PDL
ab
a1
a2
Periostin
ab
PDL
c
c
PDL
ab
b1
b2
Experiment

Figure S6

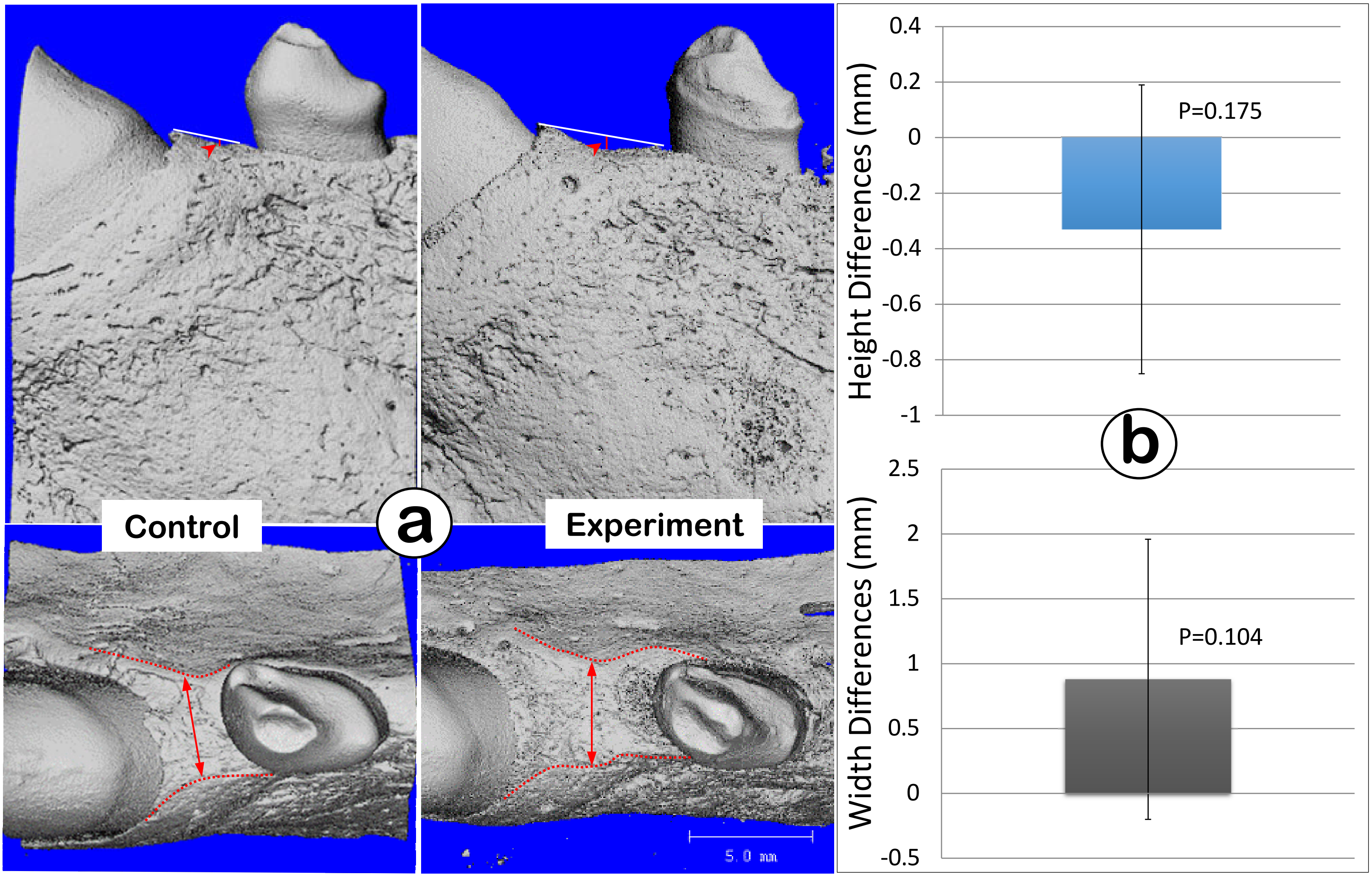

Control
a
Experiment
5.0 mm
b
Height Differences (mm)
0.4
0.2
0
-0.2
-0.4
-0.6
-0.8
-1
P=0.175
Width Differences (mm)
2.5
2
1.5
1
0.5
0
-0.5
P=0.104

Figure S7

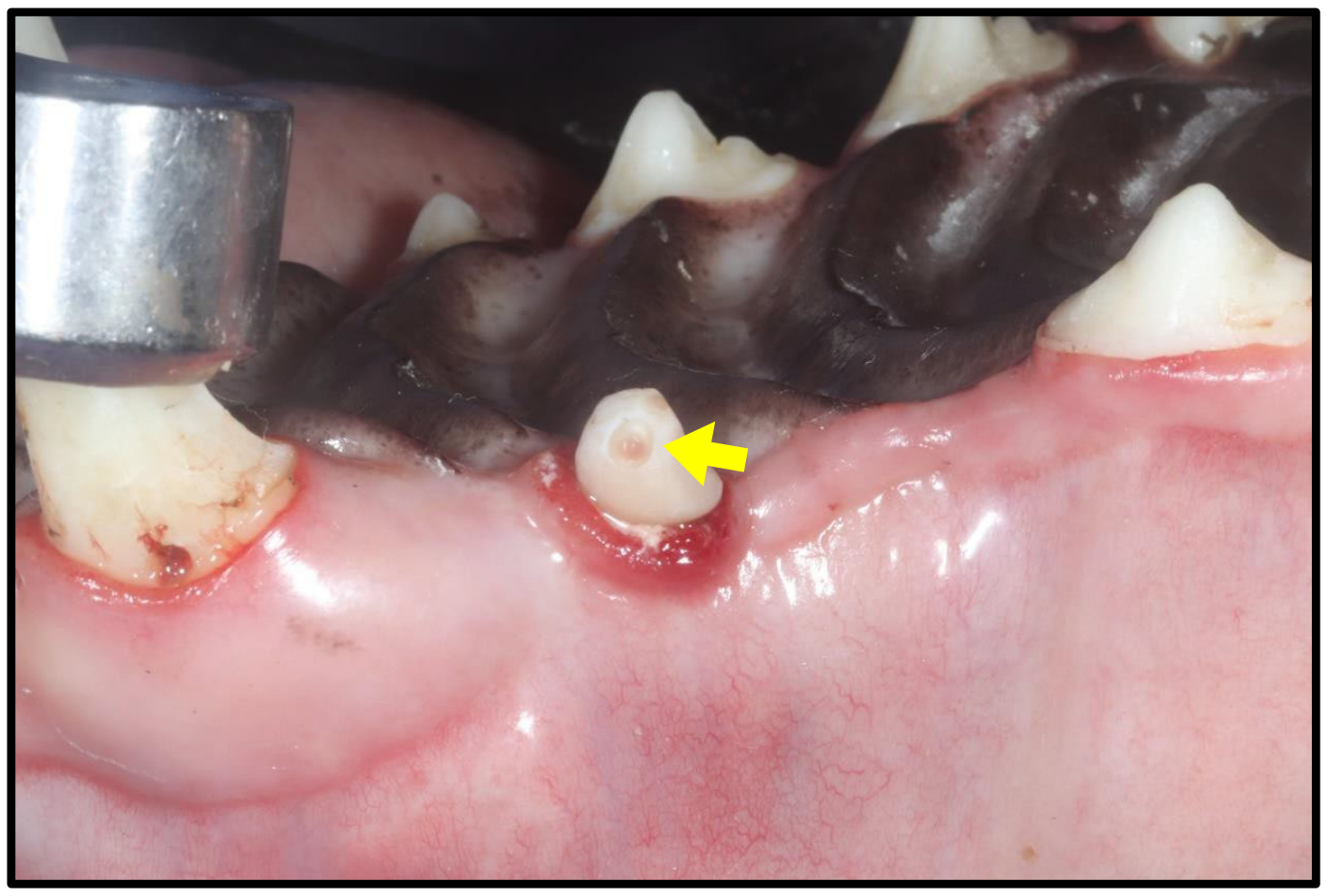

Supplement: Supplementary file 1 [file OCR-29-1006-s001.pdf]
