# Supplementary material for: The Effects of Rapid Periodontal Distraction on Tooth Vitality and Bone Quality/Quantity: A Split‐Mouth Experimental Design
Source: Orthod Craniofac Res. 2026 May 26;29(6):1006–16. doi: 10.1111/ocr.70143 (PMC13752665; doi:10.1111/ocr.70143)
Supplement: Supplementary file 2 [file OCR-29-1006-s002.docx]

Table S1: Experimental literature evaluating periodontal distraction and pulpal vitality. Only 2 animal studies have tested pulpal vitality after PD.

| Reference | mm/day | mm Tooth Movement | Time | Pulpal Vitality |
| --- | --- | --- | --- | --- |
| Ai et al^10^ | 0.5 | 3.6 | 2 weeks | N/A |
| Lv et al^11^ | 0.5 | 5.0 | 2 weeks | Histology, Teeth Vital |
| Shimojima et al^12^ | 0.25-1.0 | 0.9-3.4 | 4 days | N/A |
| Nagano et al^13^ | 0.5 | 2.0 | 4 days | LDF, Teeth Vital |

Table S2: Clinical studies evaluating the effects of PD on tooth movements, root resorption, and pulp vitality.

| Reference | mm/day | Duration | mm Tooth Movement | Root Resorption from periapicals | Pulp Vitality |
| --- | --- | --- | --- | --- | --- |
| Liou et al^9^ | 0.5-1.0 | 3 weeks | 6.5 | None to light blunting/irregular | EPT: 9/26 teeth vital >1 mo post PD |
| Sayin et al^14^ | 0.75 | 3 weeks | 3-5-5.8 | No significant root resorption of canines | N/A |
| Kumar et al^15^ | 0.5 | 20.33 days | 5.25 | Minimal | EPT: No vitality loss post-PD |
| Kharkar et al^16^ | 0 .5 | 19.5 days | N/A | 1 case minimal root resorption | EPT: No vitality loss at device removal, 6 and 12 mo post-PD |
| Mowafy et al^17^ | 0.25 | 5.3 weeks | 5.9 | N/A | N/A |
| Kumar et al^18^ | 0.4 | 3.5 weeks | 6.4 | N/A | EPT: Tested 1 month post-PD but vitality not reported |
| Khanna et al^19^ | N/A | 3 months | 6.9 | N/A | N/A |
| Leethanakul et al^20^ | N/A | 3 months | 2.85 | N/A | N/A |
| Kateel et al ^21^ | 0.8 | 15.4 days | 6.63 | N/A | EPT: 5/16 teeth vital post-PD |

Table S3: Total 1^st^ premolar movements and tipping over the 6-day experimental period

|  | Experimental | Control | Prob |
| --- | --- | --- | --- |
| Intraoral C-P1 (mm) | 4.8 ± 0.5 | 0.0 | **<0.001** |
| Cast C-P1 (mm) | 4.7 ± 0.4 | 0.02 ± 0.2 | **0.027** |
| Intraoral Mean Rate (mm) | 0.8 ± 0.1 | 0.0 | **<0.001** |
| Radiographic Tipping (°) | 12.8 ± 2.0 | 0.0 | **<0.001** |

Table S4: μCT Qualitative Assessment of the presence (X) or absence (O) of resorptive lacunae on the mesial, distal, buccal and lingual surfaces of the 1^st^ premolar roots.

|  | Mesial | Distal | Buccal | Lingual |
| --- | --- | --- | --- | --- |
| Dog B | O | O | X | X |
| Dog C | O | X | X | X |
| Dog D | O | X | X | X |
| Dog E | O | X | O | X |
| Dog F | X | X | X | X |
| Dog G | X | O | X | X |

Table S5: μCT analysis of lamina dura adjacent to the 1^st^ premolar

| Parameters | Experimental | | Control | | Difference |
| --- | --- | --- | --- | --- | --- |
|  | Mean | SD | Mean | SD | Prob |
| Bone volume/total volume | 0.51 | .06 | 0.60 | 0.11 | 0.099 |
| Apparent Density (mg HA/cm^3^) | 392.66 | 50.91 | 497.08 | 72.08 | 0.061 |
| Material Density (mg HA/cm^3^) | 682.10 | 60.68 | 760.06 | 22.60 | **0.029** |
| Trabecular Number (1/mm) | 5.93 | 1.42 | 4.97 | 0.82 | 0.205 |
| Trabecular Thickness (mm) | 0.17 | 0.02 | 0.24 | 0.04 | **0.015** |
| Trabecular Spacing (mm) | 0.21 | 0.08 | 0.21 | 0.06 | 0.970 |

Table S6: μCT analysis of the trabecular bone adjacent to the lamina dura of the 1^st^ premolar.

| Parameters | Experimental | | Control | | Difference |
| --- | --- | --- | --- | --- | --- |
|  | Mean | SD | Mean | SD | Prob |
| Bone volume/total volume | 0.47 | 0.11 | 0.49 | 0.14 | 0.781 |
| Apparent Density (mg HA/cm^3^) | 394.26 | 73.48 | 409.50 | 102.04 | 0.739 |
| Material Density (mg HA/cm^3^) | 716.33 | 25.13 | 751.21 | 24.35 | **0.005** |
| Trabecular Number (1/mm) | 4.49 | 0.98 | 4.05 | 1.44 | 0.434 |
| Trabecular Thickness (mm) | 0.18 | 0.02 | 0.20 | 0.04 | 0.185 |
| Trabecular Spacing (mm) | 0.27 | 0.07 | 0.34 | 0.13 | 0.184 |

Figure legends for supplementary figures

Figure S1: (a) Intraoral images showed before (left) and after (right) crown lengthening for the maxillary 1^st^ premolar (arrow). (b-c) Distraction device was fabricated on a stone cast (b) and cemented intraorally (c). (d) X-ray was taken to confirm that the distraction device was cemented in place.

Figure S2: (a) Root length measurement was used to determine the site of surgical cut on the buccal surface needed to visualize the root apex. (b) The maxillary 2^nd^ premolar was extracted. (c) Surgical guide was used to ensure sufficient vertical and transverse space for tooth distraction. (d) oblique undermining grooves were made to weaken the interseptal bone distobuccally and distolingually (yellow arrows).

Figure S3. A horizontal reference line (yellow) was drawn from the alveolar crest distal of the canine to the alveolar crest mesial to the 3^rd^ premolar; vertical reference lines (red) were drawn through the long axes of the 1^st^ and 3^rd^ premolars. Tipping of the 1^st^ premolar was calculated based on angular changes (red arrow) over time. Changes in 3^rd^ premolar angulation (yellow arrow) over time were used as adjustment factors for radiographic distortion.

Figure S4: IHC showed a high expression of Osterix (Osx) in the odontoblasts in the

teeth. Od: odontoblast

Figure S5. IHC images showed Periostin was evenly expressed on the compression and tension sides of control (a1, a2) and experimental teeth (b1, b2). ab: alveolar bone; c: cementum.

Figure S6. μCT imaging demonstrated that the regenerated bone exhibited comparable height (red arrow heads in the upper panel) and width (red double arrows in the lower panel) to the contralateral control side (a). Quantitative analyses confirmed that there were no statistically significant between-side differences in bone width or height (b) of post-consolidation regenerate and control bone.

Figure S7: Pulpal blushing (arrow) of one experimental tooth indicated tooth pulp vitality.
